# Supplementary material for: A Maastrichtian insect assemblage from Patagonia sheds light on arthropod diversity previous to the K/Pg event
Source: Commun Biol. 2023 Dec 11;6:1249. doi: 10.1038/s42003-023-05596-2 (PMC10711029; doi:10.1038/s42003-023-05596-2)
Supplement: Supplementary file 2 — Reporting summary [file 42003_2023_5596_MOESM2_ESM.pdf]

Corresponding author(s): FERNANDO E. NOVAS

Last updated by author(s): Nov 15, 2023

Reporting Summary

Nature Portfolio wishes to improve the reproducibility of the work that we publish. This form provides structure for consistency and transparency in reporting. For further information on Nature Portfolio policies, see our Editorial Policies and the Editorial Policy Checklist.

Statistics

For all statistical analyses, confirm that the following items are present in the figure legend, table legend, main text, or Methods section.

n/a	Confirmed	
☒	☐	The exact sample size (*n*) for each experimental group/condition, given as a discrete number and unit of measurement
☒	☐	A statement on whether measurements were taken from distinct samples or whether the same sample was measured repeatedly
☒	☐	The statistical test(s) used AND whether they are one- or two-sided<br>*Only common tests should be described solely by name; describe more complex techniques in the Methods section.*
☒	☐	A description of all covariates tested
☒	☐	A description of any assumptions or corrections, such as tests of normality and adjustment for multiple comparisons
☒	☐	A full description of the statistical parameters including central tendency (e.g. means) or other basic estimates (e.g. regression coefficient) AND variation (e.g. standard deviation) or associated estimates of uncertainty (e.g. confidence intervals)
☒	☐	For null hypothesis testing, the test statistic (e.g. F, t, r) with confidence intervals, effect sizes, degrees of freedom and P value noted<br>*Give P values as exact values whenever suitable.*
☒	☐	For Bayesian analysis, information on the choice of priors and Markov chain Monte Carlo settings
☒	☐	For hierarchical and complex designs, identification of the appropriate level for tests and full reporting of outcomes
☒	☐	Estimates of effect sizes (e.g. Cohen's *d*, Pearson's *r*), indicating how they were calculated

Our web collection on statistics for biologists contains articles on many of the points above.

Software and code

Policy information about availability of computer code

Data collection	No software was used
Data analysis	No software was used

For manuscripts utilizing custom algorithms or software that are central to the research but not yet described in published literature, software must be made available to editors and reviewers. We strongly encourage code deposition in a community repository (e.g. GitHub). See the Nature Portfolio guidelines for submitting code & software for further information.

Data

Policy information about availability of data

All manuscripts must include a data availability statement. This statement should provide the following information, where applicable:

- Accession codes, unique identifiers, or web links for publicly available datasets
- A description of any restrictions on data availability
- For clinical datasets or third party data, please ensure that the statement adheres to our policy

Arthropod materials are represented by approximately 30 specimens, deposited in the Museo Regional Padre Jesús Molina (Río Gallegos, Santa Cruz Province), under MPM-Pal 21835 (Palynological slides) acronyms

Human research participants

Policy information about studies involving human research participants and Sex and Gender in Research.

Reporting on sex and gender	Not aplicable
Population characteristics	Not aplicable
Recruitment	Not aplicable
Ethics oversight	Not aplicable

Note that full information on the approval of the study protocol must also be provided in the manuscript.

Field-specific reporting

Please select the one below that is the best fit for your research. If you are not sure, read the appropriate sections before making your selection.

☐ Life sciences ☐ Behavioural & social sciences ☒ Ecological, evolutionary & environmental sciences

For a reference copy of the document with all sections, see nature.com/documents/nr-reporting-summary-flat.pdf

Ecological, evolutionary & environmental sciences study design

All studies must disclose on these points even when the disclosure is negative.

Study description	Paleoentomological research
Research sample	Fossil arthropods collected in rock samples from Maastrichtian age beds (Chorrillo Formation), southern Patagonia, Argentina
Sampling strategy	No sample-size calculation was employed
Data collection	The discovery of chitinous remains was fortuitous, after preparing fragments of rocks with the usual techniques employed for palynology. Fossils were identified and collected by Dr. Valeria Perez Loinaze
Timing and spatial scale	Rock samples were obtained in March 2020
Data exclusions	No data were excluded
Reproducibility	Fossil samples belong and are available in paleontological collections of the Museo Padre Molina, Río Gallegos, Santa Cruz, Argentina.
Randomization	Specimens were taxonomically allocated within Arthropoda on the basis of anatomcail characteristics
Blinding	Not aplicable

Did the study involve field work? ☒ Yes ☐ No

Field work, collection and transport

Field conditions	Explored region in southern Patagonia has extreme wheather conditions (cold and rainy condition)
Location	Chorrillo Formation crops out 30km SW from El Calafate town, more precisely in La Anita farm (50°30'49.3"S 72°33' 35.9"W). Rock samples were taken around 500km high from the surface.
Access & import/export	Exploration and fossil collecting was authorized by Secretaría de Estado de Cultura de la Provincia de Santa Cruz, Argentina
Disturbance	No disturbance was produced in the task of taking rock samples

Reporting for specific materials, systems and methods

We require information from authors about some types of materials, experimental systems and methods used in many studies. Here, indicate whether each material, system or method listed is relevant to your study. If you are not sure if a list item applies to your research, read the appropriate section before selecting a response.

Materials & experimental systems

n/a	Involved in the study
☒	☐ Antibodies
☒	☐ Eukaryotic cell lines
☐	☒ Palaeontology and archaeology
☒	☐ Animals and other organisms
☒	☐ Clinical data
☒	☐ Dual use research of concern

Methods

n/a	Involved in the study
☒	☐ ChIP-seq
☒	☐ Flow cytometry
☒	☐ MRI-based neuroimaging

Palaeontology and Archaeology

Specimen provenance	Rock beds (i.e., Chorrillo Formation) were fossils were collected, crops out 30km SW from El Calafate town, more precisely in La Anita farm (50°30'49.3"S 72°33' 35.9"W). Exploration and fossil collecting was authorized by Secretaría de Estado de Cultura de la Provincia de Santa Cruz, Argentina.
Specimen deposition	Fossil samples belong and are available in paleontological collections of the Museo Padre Molina, Río Gallegos, Santa Cruz, Argentina.
Dating methods	No new dates are provided

☐ Tick this box to confirm that the raw and calibrated dates are available in the paper or in Supplementary Information.

Ethics oversight	Not ethical approval is necessary for this kind of studies

Note that full information on the approval of the study protocol must also be provided in the manuscript.
